# D- and Unnatural Amino Acid Substituted Antimicrobial Peptides With Improved Proteolytic Resistance and Their Proteolytic Degradation Characteristics

**DOI:** 10.3389/fmicb.2020.563030

**Published:** 2020-11-12

**Authors:** Jianguang Lu, Hongjiang Xu, Jianghua Xia, Jie Ma, Jun Xu, Yanan Li, Jun Feng

**Affiliations:** ^1^Key State Laboratory of Drug Innovation and Pharmaceutical Technology, China State Institute of Pharmaceutical Industry, Shanghai, China; ^2^Department of Peptide Drugs R&D, Shanghai Duomirui Biotechnology Co., Ltd., Shanghai, China; ^3^Department of Drug Evaluation and Research, Chia Tai Tianqing Pharmaceutical Group Co., Ltd., Nanjing, China; ^4^School of Pharmacy, Fudan University, Shanghai, China

**Keywords:** antimicrobial peptides, D-amino acid, unnatural amino acid, stability, antimicrobial activity, toxicity, amino acid substitution

## Abstract

The transition of antimicrobial peptides (AMPs) from the laboratory to market has been severely hindered by their instability toward proteases in biological systems. In the present study, we synthesized derivatives of the cationic AMP Pep05 (KRLFKKLLKYLRKF) by substituting L-amino acid residues with D- and unnatural amino acids, such as D-lysine, D-arginine, L-2,4-diaminobutanoic acid (Dab), L-2,3-diaminopropionic acid (Dap), L-homoarginine, 4-aminobutanoic acid (Aib), and L-thienylalanine, and evaluated their antimicrobial activities, toxicities, and stabilities toward trypsin, plasma proteases, and secreted bacterial proteases. In addition to measuring changes in the concentration of the intact peptides, LC-MS was used to identify the degradation products of the modified AMPs in the presence of trypsin and plasma proteases to determine degradation pathways and examine whether the amino acid substitutions afforded improved proteolytic resistance. The results revealed that both D- and unnatural amino acids enhanced the stabilities of the peptides toward proteases. The derivative DP06, in which all of the L-lysine and L-arginine residues were replaced by D-amino acids, displayed remarkable stability and mild toxicity *in vitro* but only slight activity and severe toxicity *in vivo*, indicating a significant difference between the *in vivo* and *in vitro* results. Unexpectedly, we found that the incorporation of a single Aib residue at the N-terminus of compound UP09 afforded remarkably enhanced plasma stability and improved activity *in vivo*. Hence, this derivative may represent a candidate AMP for further optimization, providing a new strategy for the design of novel AMPs with improved bioavailability.

## Introduction

The increasing emergence of antibiotic resistance has become one of the most serious issues currently threatening public health (Laxminarayan et al., 2016). In 2017, the World Health Organization published a priority list of antibiotic-resistant bacteria to encourage the research and development of effective drugs against these pathogens. The critical-priority bacteria included carbapenem-resistant *Acinetobacter baumannii (A. baumannii*) and *Pseudomonas aeruginosa* (*P. aeruginosa)* and carbapenem-resistant and third-generation-cephalosporin-resistant Enterobacteriaceae. The highest ranked gram-positive bacteria (high priority) were vancomycin-resistant *Enterococcus faecium* and methicillin-resistant *Staphylococcus aureus (S. aureus)* (Tacconelli et al., 2018). However, despite the great clinical demand for such drugs, the discovery and development of new antibiotics remains a major challenge. In the past 20 years, only two new classes of antibiotics have been approved for the treatment of gram-positive bacteria, namely, oxazolidinones and cyclic lipopeptides (Luepke et al., 2017). Consequently, there is an urgent need to develop new antimicrobial agents that are active against microbial pathogens and less likely to induce the development of drug resistance.

Antimicrobial peptides (AMPs) have displayed increasing promise as potential anti-infective drugs for therapeutic use owing to their rapid bactericidal capacities, structural diversity, and potent broad-spectrum activity (Lee et al., 2016). In addition to several already approved AMPs of natural origin (e.g., polymyxin, vancomycin, and daptomycin), some other natural and artificially designed AMPs are also in various stages of clinical development (Greber and Dawgul, 2017; de Breij et al., 2018).

Despite the development of numerous peptide pharmaceutical candidates, the transition of AMPs from the laboratory to the market has been greatly hindered by their rapid degradation by plasma and bacterial proteases and by fast hepatic and renal clearance, which lead to short half-lives and loss of antimicrobial activity (Mahlapuu et al., 2016). Several strategies have been employed in an effort to enhance the stability of AMPs, including the incorporation of unnatural amino acids, N- and/or C-terminal modification, cyclization, the use of non-peptidic backbones (peptidomimetics), and the multimerization of AMP monomers (Ong et al., 2014; Bartoloni et al., 2015; Khara et al., 2016; Sun et al., 2017; Zaet et al., 2017; Zhong et al., 2020).

**FIGURE 1:**
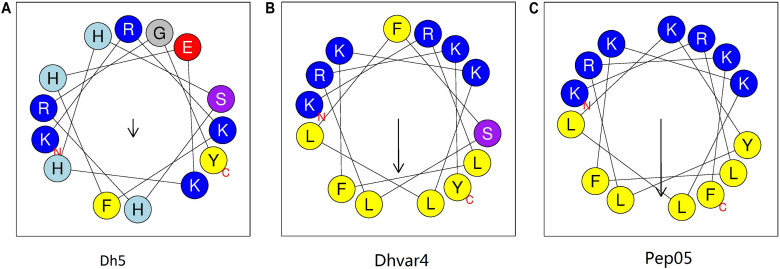
Helical wheel diagram for AMPs. The helical wheel projection was generated using the online software HeliQuest (http://heliquest.ipmc.cnrs.fr). Hydrophobic amino acids on the non-polar face of the helix are colored in yellow, and hydrophilic amino acids on the polar face of the helix are colored in blue. The length of the downward-pointing arrow indicates the strength of hydrophobicity. **(A)** dh5; **(B)** dhvar4; **(C)** Pep05. A clearer interface between the polar and non-polar faces can be seen in Pep05 compared to dh5 and dhvar4.

Histatin 5 is a 24-amino-acid peptide secreted by human salivary glands that plays a defensive role in the oral cavity (Table 1; Raj et al., 1990). The putative active domain of histatin 5 (dh5: residues 11–24) was used as a scaffold in the design of dhvar4, which has been reported to possess increased amphipathicity and enhanced potency and proteolytic resistance (Helmerhorst et al., 1999). In our previous study, we developed a cationic AMP named Pep05 (KRLFKKLLKYLRKF) based on dhvar4 (Figure 1), which exhibits high antibacterial activity and a broad activity spectrum that includes gram-negative bacteria, gram-positive bacteria, and fungi.

**TABLE 1:** Peptide sequences and physicochemical properties of histatin 5 and its derivatives.

Peptide	Sequence	Mass (Da)	<μ>^1^	<*H*>^2^	Net charge
Histatin 5	DSHAKRHHGY**KRKFHEKHHSHRGY^3^**	3,036	0.21	−0.15	+ 5
Dh5	KRKFHEKHHSHRGY	1,847	0.20	−0.17	+ 4
Dhar4	KRLFKKLLFSLRKY	1,840	0.59	0.38	+ 6
Pep05	KRLFKKLLKYLRKF	1,880	0.85	0.31	+ 7

*^1^<μ> represents the mean amphipathic moment of helicalpeptides. ^2^<*H*> represents the mean hydrophobicity of helical peptides. ^3^Bold letters represent the putative active domain of histatin 5 (residues 11 to 24).*

In the present study, we designed a series of Pep05 derivatives in which certain amino acids were replaced with structurally similar L-, D-, or unnatural amino acids and evaluated their stabilities toward trypsin, human plasma proteases, and secreted microbial proteases. To gain further insight into the degradation pathways of the substituted AMPs in the presence of various proteases and to allow subsequent targeted modifications based on the analysis residues or positions that were sensitive to proteases, the degradation products of these peptides were identified by LC-MS after treatment with trypsin or human plasma proteases. The secondary structure, hydrophobicity, antimicrobial activity, hemolytic toxicity, and cytotoxicity of the derivatives were also compared. The *in vivo* antimicrobial activity and toxicity were evaluated for two peptides, namely, DP06 (krLFkkLLkYLrkF, where k and r denote D-Lys and D-Arg, respectively) and UP09 (AibRLFKKLLKYLRKThi, where Aib and Thi denote 4-aminobutanoic acid and L-thienylalanine, respectively), which exhibited improved *in vitro* stabilities compared with Pep05 and colistin.

## Materials and Methods

### Materials

Rink amide 4-methylbenzhydrylamine resin, Fmoc-protected amino acids, peptide coupling reagents, and trifluoroacetic acid (TFA) were purchased from GL Biochem (Shanghai, China). The test strains *Escherichia coli* (*E. coli)* ATCC 25922, *P. aeruginosa* ATCC 27853, *A. baumannii* ATCC 19606, *S. aureus* ATCC 25923, and *Candida albicans* (*C. albicans*) ATCC 10231 were purchased from the American Type Culture Collection (Manassas, VA, United States). Carbapenem-resistant *P. aeruginosa* (CRPA) SIPI-012 and carbapenem-resistant *A. baumannii* (CRAB) SIPI-025 were generously gifted by Huanshan Hospital (Shanghai, China). Red blood cells and plasma were extracted from healthy blood donors. RAW 264.7 cells were obtained from the Chinese Academy of Sciences. BALB/c mice were purchased from Shanghai SIPPR-BK Laboratory Animal Co., Ltd. Trypsin, colistin, vancomycin hydrochloride, amphotericin B, 2,2,2-trifluoroethanol (TFE), and lipopolysaccharide (LPS) from *E. coli* O55:B5 were purchased from Sigma. Mueller Hinton (MH) broth, RPMI 1640 medium, Dulbecco’s modified Eagle medium (DMEM), fetal bovine serum (FBS), and 3-(4,5-dimethylthiazol-2-yl)-2,5-diphenyltetrazolium bromide (MTT) were obtained from Thermo Fisher Scientific.

### Methods

#### Peptide Synthesis

The peptides were prepared via standard Fmoc-based solid-phase synthesis on Rink amide 4-methylbenzhydrylamine resin. Diisopropylcarbodiimide and 1-hydroxybenzotriazole were used as coupling reagents, and a threefold excess of the Fmoc-amino acid was added during every coupling cycle. After cleavage and deprotection using a mixture of TFA/triisopropylsilane/H_2_O (95:2.5:2.5, v/v/v) for 3 h at room temperature, the crude peptide was repeatedly extracted with diethyl ether, dried under vacuum, and purified by RP-HPLC on a preparative Waters XBridge C18 column (length: 150 mm, internal diameter: 19 mm, pore size: 100 Å, particle size: 5 mm) using an appropriate 5–80% water/acetonitrile gradient in the presence of 0.1% TFA. The appropriate fractions were combined and lyophilized, and the final purity of the obtained peptides (> 95%) was determined by RP-HPLC on an analytical Waters XBridge C18 column (length: 250 mm, internal diameter: 4.6 mm, pore size: 100 Å, particle size: 5 mm). ESI-MS was used to determine the molecular weight.

#### Circular Dichroism Spectroscopy

The secondary structures of Pep05 and its derivatives were investigated using a Chirascan qCD Spectrometer (Applied Photophysics, United Kingdom). The peptides were dissolved to a final concentration of 0.1 mg/mL in four different solvents, namely, 10 mM phosphate buffer (PB; pH 7.4), 50% TFE/PB, 25 mM SDS in PB, and 0.1% LPS in PB (Kim et al., 2017). Circular dichroism (CD) spectra were recorded from 260 to 190 nm at 25°C as the average of three scans. Data were collected using a path length of 0.1 cm, scan speed of 50 nm/min, bandwidth of 1.0 nm, response time of 1 s, and resolution of 0.1 nm (Khara et al., 2016). Following background subtraction, the observed ellipticity [θ]_obs_ (mdeg) was converted to molar ellipticity [θ] (deg⋅cm^2^⋅dmol^–1^) using the equation [θ] = [θ]_obs_(MRW/10*cl*), where [θ]_obs_ is the observed ellipticity corrected for the blank at a given wavelength (mdeg), MRW is the mean residue molecular weight (molecular weight/number of amino acids), *c* is the peptide concentration (mg/mL), and *l* is the path length (cm). Assuming a two-state model, the percentage of α-helix structure was calculated using the following equation: α-helical content = −([θ]_222_ + 2000)/30000 × 100% (Jia et al., 2017).

#### Hydrophobicity

Each peptide was dissolved in ddH_2_O to a concentration of 0.5 mg/mL and then subjected to RP-HPLC on an analytical Waters XBridge C18 column (length: 250 mm, internal diameter: 4.6 mm) using 0.1% TFA in water as solvent A and 0.1% TFA in acetonitrile as solvent B. The peptides were eluted using a linear gradient as follows: 5%B to 45%B over 20 min, 45%B to 60%B over 2 min, and 60%B to 5%B over 1 min. The flow rate was 1 mL/min and the absorbance was measured at 215 nm. The retention time was recorded for each peptide (Kim et al., 2005).

#### Minimum Inhibitory Concentration

The antimicrobial activities of the peptides were tested against *E. coli* ATCC 25922, *P. aeruginosa* ATCC 27853, carbapenem-resistant *P. aeruginosa* SIPI-012, *A. baumannii* ATCC 19606, carbapenem-resistant *A. baumannii* SIPI-025, *S. aureus* ATCC 25923, and *C. albicans* ATCC 10231. The minimum inhibitory concentration (MIC) values were determined using the microdilution method according to Clinical and Laboratory Standards Institute guidelines (CLSI, 2008, 2012). The peptides were dissolved in 10 mM PB to a concentration of 1280 μg/mL as stock solutions.

For inoculum preparation of bacteria, morphologically similar colonies selected from fresh MH agar plates were transferred using a loop into approximately 10 mL of MH broth in a capped test tube and vortexed. The suspension was adjusted with MH broth to achieve a turbidity equivalent to a 0.5 McFarland standard (BaSO_4_ solution prepared spectrophotometrically). A working suspension was prepared by making a 1:15 dilution followed by a 1:20 dilution of the stock suspension with MH broth medium to obtain approximately 5 × 10^5^ CFU/mL. For inoculum preparation of yeast, five fresh colonies of approximately 1 mm diameter were picked from 24-hour-old cultures of *C. albicans* and suspended in 5 mL of sterile 0.85% saline. The resulting suspension was vortexed and the cell density was adjusted by adding sufficient sterile saline to achieve a turbidity equivalent to a 0.5 McFarland standard. A working suspension was prepared by making a 1:100 dilution followed by a 1:20 dilution of the stock suspension with RPMI 1640 broth medium to obtain 0.5 × 10^3^ to 2.5 × 10^3^ CFU/mL. Intermediate twofold dilutions of the peptides were prepared in MH broth and RPMI 1640 medium, and 90 μL aliquots were added to each well in addition to 10 μL of the inoculum prepared above.

Each plate included a growth control well and a sterility (uninoculated) control well. Colistin, vancomycin hydrochloride, and amphotericin B were used as positive controls for gram-negative bacteria, gram-positive bacteria, and fungi, respectively. The bacterial and fungal plates were incubated at 37°C for 20 h and 35°C for 48 h, respectively, both under ambient atmosphere. The inhibition of microbial growth was assessed visually. The MICs were determined from independent triplicate assays.

#### Stability Toward Tryptic Degradation

Each peptide was dissolved in water to a final concentration of 1 mg/mL. A trypsin solution was prepared by dissolving 1 mg of trypsin in 1 mL of 0.1 M NH_4_HCO_3_ buffer (pH 8.2). For the stability tests, 20 μL of freshly prepared trypsin solution and 80 μL of peptide solution were combined with 900 μL of 0.1 M NH_4_HCO_3_ buffer (pH 8.2) in a 2 mL Eppendorf tube and the resulting solution was incubated at 37°C on a rocking table (Svenson et al., 2008). Aliquots (200 μL) were removed after 1, 5, 18, and 24 h and diluted with an equal volume of water/acetonitrile (60:40 v/v) containing 1% TFA. The samples were analyzed as described in section “Hydrophobicity.” Take the peak area of 0 h as 100% for each peptide and the remained amounts were calculated by relative peak area monitored by HPLC. The peak area of peptide was obtained by subtraction of background peak areas in blank matrix. All stability tests were performed at least in triplicate. The identities of the degradation products after 18 h of tryptic degradation were determined by LC-MS on a UPLC-QDa system (Waters Acquity, United States).

#### Stability Toward Plasma Proteases

The peptides were mixed with plasma from healthy volunteers to a final concentration of 0.1 mg/mL and incubated at 37°C. Aliquots (20 or 40 μL) were removed after 0, 2, 4, 6, 8, 12, 24, and 36 h, mixed with 0.05% TFA to a final concentration of 0.01 mg/mL, and incubated on ice for 10 min. After centrifugation (12,000 × *g*, 5 min), the supernatant was stored at −20°C. The samples were analyzed as described in section “Hydrophobicity” and quantified as described in section “Stability Toward Tryptic Degradation.” All stability tests were performed at least in triplicate. The identities of the metabolites after 12 h of degradation by plasma proteases were determined by LC-MS on a Waters Acquity UPLC-QDa system.

To evaluate the biological activity of the degradation products, the peptides were dissolved in 10 mM PB to a concentration of 320-fold MIC and then diluted to 160-, 80-, 40-, and 20-fold MIC with MH broth. Equal volumes of plasma were added to these solutions and the mixtures were incubated at 37°C for 1 h. The MIC values of the peptides against *P. aeruginosa* ATCC 27853 were then determined as previously described.

#### Stability Toward Secreted Microbial Proteases

The stabilities of the peptides toward proteases secreted by *E. coli* ATCC 25922, *P. aeruginosa* ATCC 27853, *A. baumannii* ATCC 19606, *S. aureus* ATCC 25923, and *C. albicans* ATCC 10231 were also examined. Microbial supernatants were prepared by resuspending single colonies of each strain in 20 mL of an appropriate culture medium followed by incubation at 37°C overnight. MH broth and 2% tryptone/2% dextrose were used as the culture media for bacteria and yeast, respectively. Each culture was then centrifuged at 12,000 × *g* for 10 min and the supernatant was adjusted to pH 8.0 with HCl or NaOH and then filtered through a 0.22 μm sterile filter membrane (Zaet et al., 2017). The peptides were then dissolved in the filtered supernatants to a concentration of 0.5 mg/mL, and the peptide-free supernatants were used as negative controls. The samples were incubated at 37°C for 24 h in a water bath and then analyzed by RP-HPLC as described above.

#### Hemolytic Activity Assays

Whole blood was obtained from a healthy volunteer. The red blood cells (RBC) were then centrifuged (800 × *g*, 10 min), washed twice with PB, resuspended in 1 mL of PB, and incubated with various concentrations of the peptides (32–128 μM) at 37°C for 1 h. As a positive control, total lysis of the RBC was accomplished by incubation with 0.1% SDS. For each condition, three technical replicates were performed. After incubation, the RBC were centrifuged at 800 × *g* for 10 min and the amount of hemoglobin released into the supernatant was determined by measuring the optical density OD_420_ using a SpectraMax i3x microplate reader (Molecular Devices) (Zaet et al., 2017).

#### Cell Viability Assays

The peptides were dissolved in ddH_2_O to a concentration of 256 μM. RAW 264.7 cells were cultured in 100 μL of DMEM containing 10% FBS and seeded at a density of 1 × 10^4^ cells in a 96-well culture plate. The plate was incubated at 37°C under 5% CO_2_ atmosphere overnight. After attaining 80% confluency, the cells were treated with various concentrations of the peptides (1–128 μM) dissolved in DMEM containing 4% FBS for 42 h. Cytotoxicity was determined using the MTT tetrazolium reduction assay and quantified spectrophotometrically at 595 nm using a SpectraMax i3x microplate reader. The cell viability was calculated with respect to control samples treated only with DMEM containing 4% FBS (Khara et al., 2016). Each test was done in triplicate and the concentration required for 50% inhibition of viability (IC_50_) was determined.

#### Evaluation of *in vivo* Toxicity

A limited number of toxicity experiments were conducted using a BALB/c mouse model with a single intravenous or intraperitoneal injection of the test peptides, namely, Pep05, DP06, and UP09, at various doses from 5 to 20 mg/kg. Six-week-old mice (approximate weight: 18–20 g, SFP grade) were bred and maintained under specific pathogen-free conditions on a 12 h light/dark cycle. The peptides were dissolved in PBS (pH 7.4) and three mice were used for each dose. As a control, colistin was administered in a similar manner. The mice were monitored for 24 h and mice were weighed at the end of the experiment. The procedure was evaluated and approved by the Ethical Committee of Experimental Animals of the Chia Tai Tianqing Pharmaceutical Group in August 2019.

#### Evaluation of *in vivo* Antibacterial Activities

The *in vivo* antibacterial activities of the peptides were evaluated in BALB/c mice using *P. aeruginosa* ATCC 9027 as a test strain. A total of 20 mice (6 weeks old, approximate weight: 18–20 g, SFP grade) were first used to measure the minimal lethal doses (MLD) with 5 mice for each inoculum. Cell numbers of selected strains were determined from the colony numbers from the plate count agar and four inocula (1 × 10^6^, 5 × 10^6^, 1 × 10^7^, and 5 × 10^7^ CFU/mouse) were administered via intraperitoneal injection. The minimal lethal dose was determined by measuring the mortality rate after 72 h. BALB/c mice were then injected intraperitoneally with a lethal dose of *P. aeruginosa* ATCC 9027. Thirteen mice in each group were then administered two intraperitoneal injections (1 and 8 h post-infection) of Pep05, DP06, or UP09 (10 mg/kg), while the mice in the control group were injected with the same volume of saline. Colistin (10 mg/kg) was used as a positive control. The survival of the animals was monitored over 7 d.

## Results

### Peptide Design, Synthesis, and Characterization

Basic amino acids with net cationic charge promotes selective binding to the anionic surfaces of bacteria over the zwitterionic host cell surfaces, and hydrophobic amino acids facilitate partitioning into bacterial membranes (Kim et al., 2005; Saint Jean et al., 2018). Pep05 is a typical cationic AMP composed of basic amino acids, L-lysine (K) and L-arginine (R), and hydrophobic amino acids, L-leucine (L), L-phenylalanine (F) and L-tyrosine (Y). However, many studies have shown that the basic amino acids in peptide chains, K and R, are easily hydrolyzed by proteases in digestive tract, plasma and tissues (Svenson et al., 2008). The principle of these modifications was attempting to replace the original amino acids without causing significant changes in the physicochemical properties and biological activity compared with the parent peptide Pep05, and to determine the influence of these substitutions on the stability toward proteases. With this aim, positively charged or hydrophobic residues were replaced with structurally similar amino acids.

**FIGURE 2:**
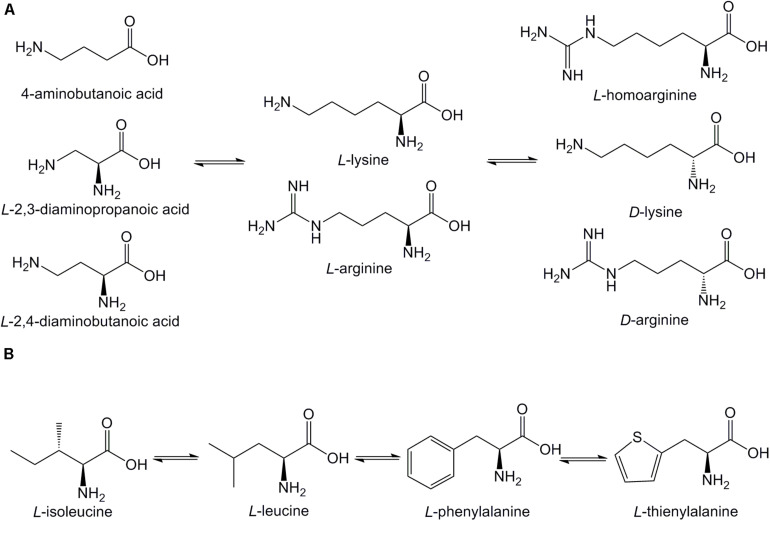
Structures of amino acids used in Pep05 derivatives. **(A)** 4-aminobutanoic acid (Aib), D-lysine (k), D-arginine (r), L-homoarginine (Hor), L-2,3-diaminopropionic acid (Dap), and L-2,4-diaminobutanoic acid (Dab) were used to replace L-lysine (K) or L-arginine (R); **(B)** L-thienylalanine (Thi) was used to replace L-phenylalanine (F).

The sequences of the synthesized Pep05 derivatives are listed in Table 2, and the structure of the amino acids used are presented in Figure 2. First, we investigated whether there were significant differences in stability between similar L-amino acids. L-Arg was used to replace L-Lys, and L-isoleucine (I) and L-Phe were used to replace L-Leu (see LP01 and LP02). Next, L-Lys and L-Arg were substituted by their D-enantiomers (k and r) in varying degrees (see DP03, DP04, DP05, and DP06). Finally, we replaced L-Lys and L-Arg with unnatural basic amino acids, such as Dab, Dap, and Hor, which possess similar structures as L-Lys and L-Arg in that they also contain amino or guanidino groups in their side chains of various lengths. Thus, the positive charge of the AMPs remained unchanged (see UP10–UP15). Thi was used to replace L-Phe since it also possesses a similar structure (see UP07, UP08). It has been reported that Aib can increase the proteolytic resistance of peptides and tends to induce a helical structure (Yamaguchi et al., 2003; Zikou et al., 2007). Thus, the N-terminal L-Lys was replaced with Aib to investigate whether this substitution could protect the peptide from proteolysis (see UP09). Successful peptide synthesis was confirmed by mass spectrometry analysis, and close agreement between the theoretical and measured molecular weights was obtained (see Supplementary Figure 1).

**TABLE 2:** Peptide sequences of Pep05 and its derivatives.

Peptides	Sequence												Theoretical MW. (Da)	Measured MW.(Da)	RT^1^ (min)
N→C	1	23	4	5	67	8	9	10	11	12	13	14			
Pep05	K	RL	F	K	KL	L	K	Y	L	R	K	F	1880.22	1880.00	15.362
LP01	K	RL	F	K	RL	F	K	Y	L	R	R	F	1970.22	1970.52	15.135
LP02	R	RI	F	K	RL	F	K	Y	L	R	R	F	1998.23	1998.60	14.937
DP03	K	RL	F	K	KL	L	K	Y	L	R	k	F	1880.22	1880.50	14.603
DP04	K	RL	F	k	kL	L	K	Y	L	R	k	F	1880.22	1880.50	14.819
DP05	k	rL	F	k	kL	L	K	Y	L	R	k	F	1880.22	1880.55	13.663
DP06	k	rL	F	k	kL	L	k	Y	L	r	k	F	1880.22	1880.55	12.425
UP07	K	RL	Thi	K	KL	L	K	Y	L	R	K	Thi	1892.26	1892.55	14.813
UP08	K	RL	F	K	KL	L	K	Y	L	R	K	Thi	1886.24	1886.60	15.161
UP09	Aib	RL	F	K	KL	L	K	Y	L	R	K	Thi	1843.17	1843.36	16.606
UP10	Hor	RL	F	K	KL	L	K	Y	L	R	K	Thi	1928.28	1928.65	15.371
UP11	Dap	HorL	F	Dap	DapL	L	Dab	Y	L	Hor	Dab	Thi	1731.94	1731.58	14.898
UP12	Dab	HorL	F	Dab	DabL	L	Dab	Y	L	R	Dab	F	1753.95	1753.62	14.393
UP13	Dab	HorL	F	Dab	DabL	L	Dab	Y	L	Hor	Dab	F	1767.98	1768.08	14.536
UP14	Dap	HorL	F	Dap	DapL	L	Dab	Y	L	Hor	Dab	F	1725.92	1725.75	15.261
UP15	Dab	RL	Thi	Dap	DabL	L	Dab	Y	L	Hor	Dab	F	1745.96	1746.08	14.157

*k, D-lysine; r, D-arginine; Thi, L-thienylalanine; Aib, L-4-aminobutanoic acid; Hor, L-homoarginine; Dab, L-2,4-diaminobutanoic acid; Dap, L-2,3-diaminopropionic acid. ^1^RP-HPLC analysis was used to assay the retention time of each peptide based on acetonitrile/water/TFA system and C18 column.*

### Peptide Secondary Structure

**FIGURE 3:**
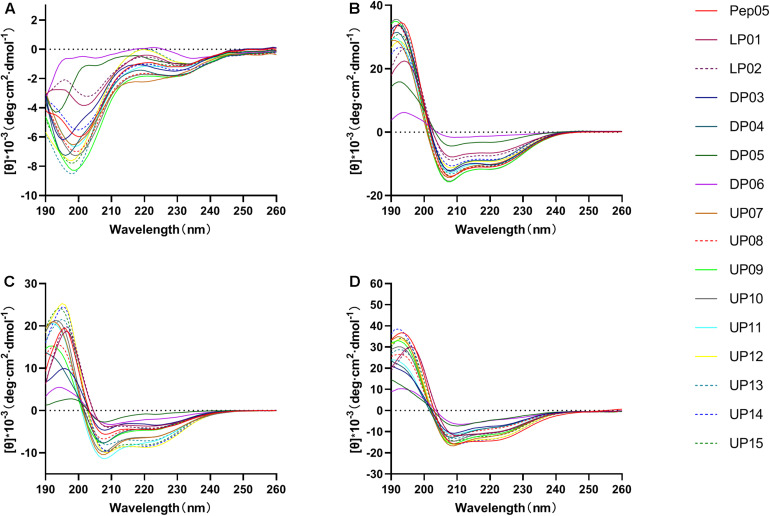
The CD spectra of Pep05 and its derivatives. The peptides were dissolved in **(A)** 10 mM PB buffer (pH 7.4), **(B)** 50% TFE, **(C)** 25 mM SDS, or **(D)** 0.1% LPS. The mean residue ellipticity was plotted against wavelength. The values from three scans were averaged per sample.

The influence of the amino acid substitutions on the peptide secondary structure was investigated using CD spectroscopy under different conditions, including 10 mM PB at pH 7.4 (mimicking an aqueous environment), 50% TFE (mimicking a hydrophobic bacterial membrane environment), 25 mM SDS (mimicking a negatively charged bacterial membrane environment), and 0.1% LPS (mimicking a bacterial outer membrane environment). The CD spectra of the peptides are presented in Figure 3 and the α-helical contents (%) of each peptide are listed in Table 3. The CD spectra indicated that all peptides adopted a definite random-coil conformation in PB. In contrast, all of the peptides except DP05 and DP06 exhibited a distinct positive peak at 192 nm and two negative peaks at 208 and 222 nm in 50% TFE, 25 mM SDS, and 0.1% LPS, which indicates a predominantly α-helical conformation. The replacement of residues with unnatural amino acids (Aib, Dab, Dap, Hor, and Thi) or a relatively small number of D-amino acids (only one D-amino acid in DP03 and three in DP04) did not compromise the helical structures of the peptides, as demonstrated by their comparable relative helicity and mean residue ellipticity values at 222 nm ([θ]_222_). While, increasing the number of D-amino acids (to five in DP05 and seven in DP06) significantly reduced the helicity relative to that of Pep05, and both DP05 and DP06 transformed into random coils in 50% TFE and 25 mM SDS.

**TABLE 3:** Mean residual ellipticity at 222 nm ([θ]_222_) and percent α-helical contents of Pep05 and its derivatives.

Peptide	PB buffer		50% TFE		25 mM SDS		0.1% LPS	
	[θ]_222_	%α-helix	[θ]_222_	%α-helix	[θ]_222_	%α-helix	[θ]_222_	%α-helix
Pep05	–908.9	rc	–10732.0	29.1	–4505.5	8.4	–14367.6	41.2
LP01	–422.2	rc	–6416.4	14.7	–3657.6	5.5	–10219.1	27.4
LP02	–557.2	rc	–7222.6	17.4	–4127.2	7.1	–10435.3	28.1
DP03	–1212.3	rc	–10161.9	27.2	–3368.4	4.6	–7277.7	17.6
DP04	–1584.2	rc	–8766.2	22.6	–4429.9	8.1	–7904.2	19.7
DP05	–705.6	rc	–3094.8	3.6	–840.7	rc	–4247.5	7.5
DP06	110.1	rc	–1132.5	rc	–2067.4	rc	–4419.9	8.1
UP07	–2177.7	rc	–9861.3	26.2	–6325.3	14.4	–11741.6	32.5
UP08	–1681.0	rc	–10685.7	29.0	–4325.1	7.8	–8456.9	21.5
UP09	–1842.7	rc	–11436.9	31.5	–4643.3	8.8	–11064.1	30.2
UP10	–1722.9	rc	–11522.2	31.7	–6296.8	14.3	–10090.1	27.0
UP11	–1148.3	rc	–10034.6	26.8	–7399.2	18.0	–7813.8	19.4
UP12	–122.8	rc	–9133.2	23.8	–8653.2	22.2	–13147.4	37.2
UP13	–39.9	rc	–10591.5	28.6	–7098.6	17.0	–10351.2	27.8
UP14	–1041.1	rc	–8543.7	21.8	–8302.1	21.0	–13227.5	37.4
UP15	–1016.6	rc	–10309.7	27.7	–8009.7	20.0	–11809.7	32.7

*% α-helix = –([θ]_222_ + 2000)/30000 × 100%. rc means random coli.*

### Hydrophobicity

**FIGURE 4:**
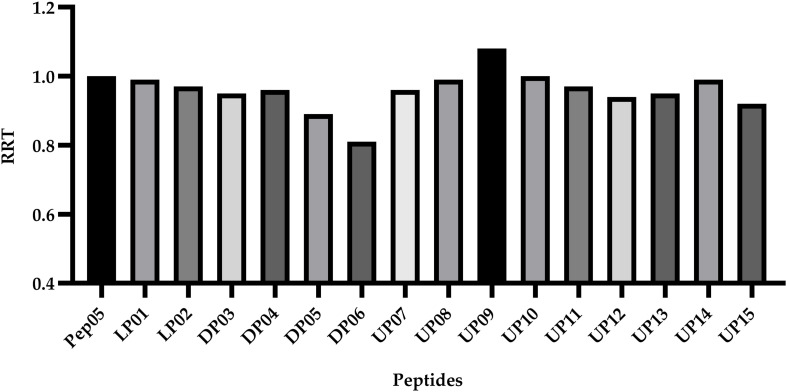
The relative retention time (RRT) calculated by comparison of the HPLC retention time of each peptide to Pep05. The RP-HPLC retention time of each peptide was used to calculate the relative retention time (RRT) with respect to Pep05. No significant changes were observed for the synthetic derivatives (0.9–1.0), with the exception of DP05 (0.89), DP06 (0.81), and UP09 (1.08).

Peptide hydrophobicity, net charge, amphipathic nature, and secondary structure are believed to be important for antimicrobial activity (Lee et al., 2016; Li J. et al., 2017). Among these, hydrophobicity has been recognized as an important factor to promote hydrophobic interaction with the hydrophobic core of the lipid bilayer, leading to membrane disruption, cytoplasm leakage, and eventual cell death (Schmidtchen et al., 2014). However, the actual hydrophobicity in liquid environment has been reported to usually show disagreement with the theoretical mean hydrophobicity (Kim et al., 2005). Peptides with different amino acid compositions and chain lengths usually possess different levels of hydrophobicity, resulting in different retention behavior during RP-HPLC. More hydrophobic compounds are typically eluted later from the column. The acetonitrile/water/TFA system was selected because this is the most widely used system for the RP-HPLC analysis of peptides owing to its superior separation efficiency compared to other mobile phases (Tripet et al., 2007). The RP-HPLC retention time of each peptide was recorded (Table 2) and used to calculate the relative retention time (RRT) with respect to Pep05 (Figure 4). No significant changes were observed for the synthetic derivatives (0.9–1.0), with the exception of DP05 (0.89), DP06 (0.81), and UP09 (1.08). These results indicate that substitutions with structurally similar amino acids without any change in peptide length had little effect on hydrophobicity of the derivative. Compared to DP03 and DP04, DP05 and DP06 containing more D-amino acid substitutions exhibited decreased hydrophobicity, suggesting a negative correlation between the number of D-amino acid substitutions and hydrophobicity. It can be seen from the CD results that the introduction of D-amino acids led to decreased helicity and alteration of the spatial structure, which presumably accounted for the change in hydrophobicity. Similar findings have been reported in previous studies (Lee et al., 2004; Huang et al., 2014). As Aib contains one amino group less than L-Lys, the replacement of L-Lys with Aib in UP09 led to greater hydrophobicity.

### Antimicrobial Activities *in vitro*

The MIC values of each peptide against gram-positive bacteria, gram-negative bacteria, fungi, and clinical isolates of carbapenem-resistant *P. aeruginosa* SIPI-012 and *A. baumannii* SIPI-025 were measured. The results are summarized in Table 4. Overall, substitution with structurally similar amino acids retained the antimicrobial activities. A range of MIC values from 0.5- to 2-fold was observed for the modified peptides compared with Pep05. Furthermore, all of the derivatives displayed similar MIC values as Pep05 against the carbapenem-resistant clinical isolates of *P. aeruginosa* and *A. baumannii*. Despite being originally derived from the antifungal peptide histatin 5, Pep05 and its analogs exhibit the typical characteristics of α-helical cationic AMPs, and the MIC values revealed that they also still retained antifungal activities. These results indicated that cationic AMPs can act not only on bacterial membranes but also on fungi, as in the case of the peptide polybia-MPI (Wang et al., 2014).

**TABLE 4:** The MICs of Pep05 and the derivatives against standard strains.

Peptides	MIC (μg/mL)						
	*E. coli* ATCC 25922	*P. aeruginosa* ATCC 27853	CRPA SIPI-012	*A. baumannii* ATCC 19606	CRAB SIPI-025	*S. aureus* ATCC 25923	*C. albicans* ATCC 10231
Pep05	1	2	4	2	2	1	4
LP01	2	1	4	2	2	1	4
LP02	2	2	8	2	4	1	8
DP03	1	1	8	2	2	1	4
DP04	1	1	4	2	1	0.5	4
DP05	2	1	8	1	2	1	2
DP06	1	1	8	1	2	0.5	2
UP07	1	1	4	2	2	1	4
UP08	2	2	4	2	2	1	4
UP09	2	2	4	2	2	1	4
UP10	2	1	4	2	2	0.5	4
UP11	2	2	8	2	2	1	4
UP12	2	2	4	2	4	1	4
UP13	2	2	4	2	2	0.5	4
UP14	2	2	8	4	4	0.5	4
UP15	2	1	4	2	2	1	4
Colistin	0.25	0.5	1	0.25	0.25	/	/
Van	/	/	/	/	/	0.5	/
AmB	/	/	/	/	/	/	0.25

*CRPA SIPI-012 represents clinical isolate of carbapenem-resistant *P. aeruginosa*. CRAB SIPI-025 represents clinical isolate of carbapenem-resistant *A. baumannii*. Van, vancomycin hydrochloride; AmB, Amphotericin B. “/” represents “not suitable for this test”.*

### Stability Toward Tryptic Degradation

Trypsin is one of the major proteolytic enzymes in the gastrointestinal tract and cleaves peptides at the C-terminal side of the positively charged residues L-Lys and L-Arg. The stabilities of Pep05 and its derivatives in the presence of trypsin were measured by HPLC. As shown in Table 5 and Supplementary Figure 2, the majority of peptides that contained L-Lys and L-Arg were very susceptible to trypsin and underwent complete degradation in 1 h (e.g., Pep05, LP01, LP02, DP03, DP04, DP05, UP07, UP08, UP09, and UP10). UP12 and UP15, both of which contained only one L-Arg residue located at the head and tail region of the peptide chain, respectively, displayed different susceptibilities to trypsin. UP12 was entirely degraded in 1 h, whereas 60% of UP15 remained, indicating that the local peptide microenvironment may significantly influence the hydrolysis of trypsin to a specific substrate. UP11, UP13, and UP14, in which all of the L-Lys and L-Arg residues were substituted by Dab, Dap, or Hor, exhibited significantly improved stability to trypsin, with at least 40% remaining after 5 h. Most notably, DP06, with all of the L-Lys and L-Arg residues substituted by D-amino acids, exhibited the highest resistance to trypsin, with 15% still remaining after 18 h and 6% after 24 h, respectively.

**TABLE 5:** The residual amounts of Pep05 derivatives at different time intervals following trypsin incubation.

Peptides	Relative peak area (%)^1^			
	1 h	5 h	18 h	24 h
DP06	82.99 ± 1.41	40.89 ± 1.89	15.60 ± 1.52	6.36 ± 1.50
UP11	87.39 ± 3.05	47.43 ± 3.82	∼0	ND^2^
UP13	95.38 ± 0.88	38.22 ± 1.62	0.66 ± 0.16	ND
UP14	74.61 ± 3.25	61.12 ± 3.39	1.37 ± 0.41	ND
UP15	59.46 ± 2.97	19.34 ± 1.65	4.36 ± 0.37	ND

*^1^Take the peak area of 0 h as 100% for each peptide and the remained amounts were calculated by relative peak area monitored by HPLC. The peak area of peptide was obtained by subtraction of background peak areas in blank matrix. Results represent the mean (± standard deviation SD). Each experiment was performed in triplicate. ^2^ND means “not determined”.*

### Analysis of Tryptic Degradation Products

To elucidate the degradation characteristics of substituted AMPs in the presence of trypsin, the degradation products of each peptide were analyzed by LC-MS. The molecular weights of major fragments retained on the column were measured to determine the tryptic cleavage sites. Single cleaved amino acid residues and dipeptides, such as L-Lys, L-Arg, KR, and RR, were difficult to detect owing to their strong polarity and thus limited retention in RP-HPLC. As shown in Table 6, cleavage occurred at the C-terminal sides of almost all of the L-Lys and L-Arg residues in the peptides, as expected, to afford smaller peptide fragments or individual amino acids. The replacement of L-Lys and L-Arg with D-Lys, D-Arg, L-Dab, L-Dap, or L-Hor significantly protected the peptides from degradation as these unnatural residues are not ideal substrates for trypsin. Compared with peptides containing L-Lys and L-Arg, the peptides in which all of the L-Lys and L-Arg residues had been replaced with D- or unnatural amino acids (DP06, UP11, UP13, and UP14) were only partially degraded and the major degradation products were larger fragments.

**TABLE 6:** Identification of tryptic degradation fragments of peptides by LC-MS.

MW.^1^	Fragment^2^	Location	MW.	Fragment	Location
**Pep05**	**KRLFKKLLKYLRKF**		500.40	(K) KLLK (Y)	6–9
406.30	(R) LFK (K)	3–5	372.30	(K) LLK (Y)	7–9
372.27	(K) LLK (Y)	7–9	450.32	(K) YLR (K)	10–12
450.31	(K) YLR (K)	10–12	412.24	(R) LThiK (K)	3–5
500.39	(K) KLLK (Y)	6–9	**UP08**	**KRLFKKLLKYLRKThi**	
534.42	(R) LFKK (L)	3–6	170.11	(K) Thi	14
**LP01**	**KRLFKRLFKYLRRF**		500.40	(K) KLLK (Y)	6–9
320.18	(R) RF	13–14	534.44	(R)LFKK (L)	3–6
606.38	(K) YLRR (F)	10–13	372.30	(K) LLK (Y)	7–9
164.16	(R) F	14	450.32	(K) YLR (K)	10–12
450.29	(K) YLR (R)	10–12	406.26	(R) LFK (K)	3–5
562.80	(K) RLFK (R)	2–5, 6–9	**UP09**	**AibRLFKKLLKYLRKThi**	
562.80	(R) LFKR (L)	3–6	500.35	(K) KLLK (Y)	6–9
406.29	(R) LFK (R)	3–5	534.38	(R) LFKK (L)	3–6
406.29	(R) LFK (Y)	7–9	372.26	(K) LLK (Y)	7–9
**LP02**	**RRIFKRLFKYLRRF**		450.29	(K) YLR (K)	10–12
320.14	(R) RF	13–14	406.29	(R) LFK (K)	3–5
606.44	(K) YLRR (F)	10–13	**UP10**	**HorRLFKKLLKYLRKThi**	
164.16	(R) F	14	500.37	(K) KLLK (Y)	6–9
562.40	(R) RIFK (R)	2–5	534.54	(R) LFKK (L)	3–6
450.28	(K) YLR (R)	10–12	372.25	(K) LLK (Y)	7–9
406.30	(R) IFK (R)	3–5	450.29	(K) YLR (K)	10–12
562.40	(R) IFKR (L)	3–6	406.28	(R) LFK (K)	3–5
406.30	(R) LFK (Y)	7–9	**UP11**	**DapHorLFDapDapLLDabYLHorDabThi**	
**DP03**	**KRLFKKLLKYLRkF**		534.35	(Dap) HorLFDap (Dap)	2–5
500.38	(K) KLLK (Y)	6–9	1196.38	DapHorLFDapDapLLDab (Y)	1–9
534.35	(R) LFKK (L)	3–6	1479.55	DapHorLFDapDapLLDabYLHor (Dab)	1–12
372.27	(K) LLK (Y)	7–9	1645.62	(Dap) HorLFDapDapLLDabYLHorDabThi	2–14
450.31	(K) YLR (k)	10–12	1731.72	DapHorLFDapDapLLDabYLHorDabThi	1–14
406.30	(R) LFK (K)	3–5	**UP12**	**DabHorLFDabDabLLDabYLRDabF**	
724.80	(K) YLRkF	10–14	550.44	(Dab) YLRDab (F)	10–13
**DP04**	**KRLFkkLLKYLRkF**		976.82	(Dab) DabLLDabYLRDab (F)	6–13
500.38	(k) kLLK (Y)	6–9	1238.58	(Hor) LFDabDabLLDabYLR (Dab)	3–12
690.90	KRLFk (k)	1–5	974.98	(Dab) HorLFDabDabLLDab (Y)	2–9
690.90	(K) RLFkk (L)	2–6	1507.74	DabHorLFDabDabLLDabYLR (Dab)	1–12
372.27	(K) LLK (Y)	7–9	**UP13**	**DabHorLFDabDabLLDabYLHorDabF**	
450.31	(K) YLR (k)	10–12	547.40	(Dab) HorLFDab (Dab)	2–5
724.80	(K) YLRkF	10–14	974.88	(Dab) HorLFDabDabLLDab (Y)	2–9
562.37	(K) RLFk (k)	2–5	1521.30	DabHorLFDabDabLLDabYLHor (Dab)	1–12
**DP05**	**krLFkkLLKYLRkF**		1521.30	(Dab) HorLFDabDabLLDabYLHorDab (F)	2–13
450.29	(K) YLR (k)	10–12	1767.74	DabHorLFDabDabLLDabYLHorDabF	1–14
1173.50	krLFkkLLK (Y)	1–9	**UP14**	**DapHorLFDapDapLLDabYLHorDabF**	
724.50	(K) YLRkF	10–14	534.35	(Dap) HorLFDab (Dab)	2–5
**DP06**	**krLFkkLLkYLrkF**		1196.28	DapHorLFDapDapLLDab (Y)	1–9
1605.84	krLFkkLLkYLr (k)	1–12	1479.84	DapHorLFDapDapLLDabYLHor (Dab)	1–12
1595.74	(r) LFKdkLLkYLrkF	3–14	1725.70	DapHorLFDapDapLLDabYLHorDabF	1–14
1321.62	(r) LFkkLLkYLr (k)	3–12	**UP15**	**DabRLThiDapDabLLDabYLHorDabF**	
1880.20	krLFkkLLkYLrkF	1–14	952.90	(Dab) RLThiDapDabLLDab (Y)	2–9
**UP07**	**KRLThiKKLLKYLRKThi**		1243.08	(R) LThiDapDabLLDabYLHor (Dab)	3–12
170.11	(K) Thi	14	1489.44	(R) LThiDapDabLLDabYLHorDab (F)	3–14
540.33	(R) LThiKK (L)	3–6	1745.68	DabRLThiDapDabLLDabYLHorDabF	1–14

*^1^The molecular weights of some fragments were calculated from a series of multiply charged ions. ^2^Fragments were produced when peptides were cleaved between the residues in the brackets and out of the brackets.*

### Stability Toward Human Plasma Proteases

#### Degradation in Human Plasma Measured by HPLC

**FIGURE 5:**
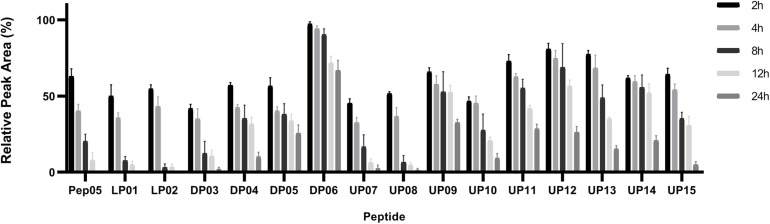
Stabilities of Pep05 and its derivatives in human plasma, as determined by HPLC. The peptides were mixed with plasma to a final concentration of 0.1 mg/mL and incubated at 37°C. Aliquots were removed at different time interval and analyzed by HPLC. Take the peak area of 0 h as 100% for each peptide and the remained amounts were calculated by relative peak area monitored by HPLC. The peak area of peptide was obtained by subtraction of background peak areas in blank matrix. All stability tests were performed at least in triplicate.

Peptides composed entirely of L-amino acids or with only a small number of D- and unnatural amino acids, such as Pep05, LP01, LP02, DP03, DP04, DP05, UP07, UP08, and UP10, underwent over 40% degradation in 2 h and more than 50% degradation in 4 h in plasma, and almost complete degradation was observed after 12 h for Pep05, LP01, LP02, UP07, and UP08 (Figure 5). UP11, UP12, UP13, and UP14 displayed significantly superior stability compared to Pep05 after replacement of the L-Lys and L-Arg residues with L-Dab, L-Dap, or L-Hor. Most notably, DP06 exhibited the greatest stability among all of the peptides to plasma proteases, with more than 90% and 60% of the original amount of peptide remaining after 8 and 24 h, respectively. The results suggested that the peptide stability increased as the number of L-Lys and L-Arg residues replaced with D- and unnatural amino acids increased. However, rather differently from the aforementioned peptides, we were surprised to find that UP09 also displayed improved stability to plasma proteases, with over 50% and over 30% of the original amount remaining after 12 and 24 h, respectively. The superior stability can probably be ascribed to the replacement of the N-terminal L-Lys residue with Aib rather than replacement of the C-terminal L-Phe residue with Thi since UP07, UP08 and UP10 which also had a Thi residue at the same location and displayed much weaker stability. These results indicated that an N-terminal Aib residue could effectively protect these peptides from degradation by plasma proteases.

#### Antimicrobial Activities of Plasma Degradation Products

To further examine the influence of human plasma proteases on the antimicrobial activities of the peptides, the MIC values of the peptides after incubation with human plasma were determined. As some of the components of human plasma samples precipitate during incubation with MH broth, it is difficult to clearly distinguish between these white plasma precipitates and grown microorganisms. Therefore, *P. aeruginosa* ATCC 27853 was chosen as the control strain because the color of suspensions of this bacteria turns green after growth. DP06 did not exhibit any change in the MIC values after pre-incubation in 50% plasma for 1 h and incubation in 10% plasma for 16 h. In contrast, DP05 and UP13 displayed a twofold increase in the MIC value, while all the other peptides showed a 4–8-fold increase (Supplementary Figure 3). These observations were essentially consistent with the RP-HPLC results. The reason for the incomplete loss of the activity of the AMPs is that they were first degraded into large fragments in the early stage of plasma incubation, as shown in Table 7. As these fragments still possess a certain length, positive charge, and amphiphilic characteristics, and thus, they retained partial antibacterial activities. Although the corresponding fragments were not synthesized to evaluate the activities of these peptides, our previous studies have shown that residues 4–14 of Pep05 retain comparable activity to intact Pep05 despite the removal of the three N-terminal residues. These data clearly demonstrate that both D- and unnatural residues contribute to resistance to plasma proteases.

**TABLE 7:** Identification of plasma proteolytic degradation fragments of peptides by LC-MS.

MW.^1^	Fragment^2^	Location	MW.	Fragment	Location
**Pep05**	**KRLFKKLLKYLRKF**		1764.70	(K) RLThiKKLLKYLRKThi	2–14
1596.64	(R) LFKKLLKYLRKF	3–14	1892.52	KRLThiKKLLKYLRKThi	1–14
1752.15	(K) RLFKKLLKYLRKF	2–14	**UP08**	**KRLFKKLLKYLRKThi**	
1880.20	KRLFKKLLKYLRKF	1–14	1488.56	(L) FKKLLKYLRKThi	4–14
**LP01**	**KRLFKRLFKYLRRF**		1886.52	KRLFKKLLKYLRKThi	1–14
293.17	(L) FK (R)	4–5	**UP09**	**AibRLFKKLLKYLRKThi**	
293.17	(L) FK (Y)	8–9	1601.68	(R) LFKKLLKYLRKThi	3–14
752.88	(K) YLRRF	10–14	1843.00	AibRLFKKLLKYLRKThi	1–14
1842.28	(K) RLFKRLFKYLRRF	2–14	**UP10**	**HorRLFKKLLKYLRKThi**	
1970.40	KRLFKRLFKYLRRF	1–14	1601.50	(R) LFKKLLKYLRKThi	3–14
**LP02**	**RRIFKRLFKYLRRF**		1757.90	(Hor) RLFKKLLKYLRKThi	2–14
1687.05	(R) IFKRLFKYLRRF	3–14	1928.65	HorRLFKKLLKYLRKThi	1–14
1841.75	(R) RIFKRLFKYLRRF	2–14	**UP11**	**DapHorLFDapDapLLDabYLHorDabThi**	
1998.60	RRIFKRLFKYLRRF	1–14	1645.70	(Dap) HorLFDapDapLLDabYLHorDabThi	2–14
**DP03**	**KRLFKKLLKYLRkF**		1731.58	DapHorLFDapDapLLDabYLHorDabThi	1–14
562.49	(K) RLFK(K)	2–5	**UP12**	**DabHorLFDabDabLLDabYLRDabF**	
1449.22	KRLFKKLLKYL (R)	1–11	1483.68	(Hor) LFDabDabLLDabYLRDabF	
1753.56	(K) RLFKKLLKYLRkF	2–14	1654.15	(Dab) HorLFDabDabLLDabYLRDabF	3–14
1880.20	KRLFKKLLKYLRkF	1–14	1753.95	DabHorLFDabDabLLDabYLRDabF	1–14
**DP04**	**KRLFkkLLKYLRkF**		**UP13**	**DabHorLFDabDabLLDabYLHorDabF**	
562.58	KRLF (k)	1–4	1497.92	(Hor) LFDabDabLLDabYLHorDabF	3–14
562.58	(Y) LRkF	11–14	1668.25	(Dab) HorLFDabDabLLDabYLHorDabF	2–14
1880.25	KRLFkkLLKYLRkF	1–14	1767.85	DabHorLFDabDabLLDabYLHorDabF	1–14
**DP05**	**krLFkkLLKYLRkF**		**UP14**	**DapHorLFDapDapLLDabYLHorDabF**	
725.64	(K) YLRkF	10–14	1473.80	(Hor) LFDapDapLLDabYLHorDabF	3–14
1045.28	krLFkkLL (K)	1–8	1640.30	(Dap) HorLFDapDapLLDabYLHorDabF	2–14
1880.22	krLFkkLLKYLRkF	1–14	1726.00	DabHorLFDabDabLLDabYLHorDabF	1–14
**DP06**	**krLFkkLLkYLrkF**		**UP15**	**DabRLThiDapDabLLDabYLHorDabF**	
1880.22	krLFkkLLkYLrkF	1–14	1489.92	(R) LThiDapDabLLDabYLHorDab F	3–14
**UP07**	**KRLThiKKLLKYLRKThi**		1646.10	(Dab) RLThiDapDabLLDabYLHorDabF	2–14
1607.98	(R) LThiKKLLKYLRKThi	3–14	1745.60	DabRLThiDapDabLLDabYLHorDabF	1–14

*^1^The molecular weight of some fragments were calculated by a series of multiply charged ions. ^2^Fragments were produced when peptides were cleaved between the residues in the brackets and out of the brackets.*

### Analysis of Plasma Degradation Products

Although the antimicrobial activities of the peptides decreased after incubation with plasma, complete loss of activity was not observed even after almost complete degradation of the peptide, indicating that the degradation products also possessed antimicrobial activity. To determine the main degradation pathways of these peptides in the presence of plasma proteases, the major products were analyzed by LC-MS. As shown in Table 7, in contrast to the case with trypsin, the main degradation products after incubation with the plasma proteases were those in which hydrolysis had occurred at the N-terminal sides of the positively charged amino acids, such as L-Lys, L-Arg, Dab, Dap, and Hor, and hydrolysis at hydrophobic amino acids such as L-Leu and L-Tyr were also observed. As reported previously, calcium- and vitamin-K-dependent serine proteases in plasma mediate trypsin-like proteolysis, i.e., cleave C-terminally to Lys or Arg residues in the P1 position of certain sequence motifs (Karstad et al., 2012). In addition, the chymotrypsin-like protease in human plasma preferentially cleaves peptide bonds where the amino acid on the N-terminal side of the scissile amide bond (the P1 position) is a large hydrophobic amino acid (e.g., tyrosine, tryptophan, phenylalanine, or leucine) (Wang et al., 2010). No obvious degradation products of DP06 were detected after 12 h, which is consistent with the HPLC and MIC results described above.

### Stability Toward Secreted Microbial Proteases

It is worth noting that microorganisms are known to develop resistance to both conventional antibiotics and AMPs. One of the key mechanisms underlying the latter is the proteolytic degradation of AMPs (Stromstedt et al., 2009). As summarized in Table 8, each peptide was found to display distinct stabilities in the supernatants from different microorganisms, while the secreted proteases from each microorganism exhibited varying hydrolytic activities toward different peptides. Peptides composed predominantly of natural L-amino acids, such as Pep05, LP01, LP02, UP07, UP08, UP09, and UP10, underwent complete degradation in the supernatants from all six strains. In contrast, the effects of different substitutions with D- and unnatural amino acids on the susceptibility of peptides to the secreted proteases varied greatly. For example, peptides such as DP03, DP04, UP11, UP12, UP13, and UP15 displayed increased stabilities against the secreted proteases from *E. coli* and *S. aureus* but showed no improvement against those from *P. aeruginosa* and *A. baumannii*, highlighting the diversity of proteases secreted by different microorganisms. Nevertheless, DP06 still exhibited significantly improved stability in all of the bacterial and fungal supernatants tested, with more than 60% of the original peptide remaining after 24 h (Supplementary Figure 4). UP14 also displayed increased stability toward all of the secreted microbial proteases.

**TABLE 8:** Stability of Pep05 derivatives toward secreted microbial proteases.

Peptides	Percent of remained peptide amount after incubation (%)^1^				
	*E. coli* ATCC 25922	*P. aeruginosa* ATCC 27853	*A. baumannii* ATCC 19606	*S. aureus* ATCC 25923	*C. albicans* ATCC 10231
Pep05	1.370.82	/	/	22.21 ± 4.47	/
LP01	/	/	/	33.27 ± 5.69*	/
LP02	2.681.99	/	/	19.73 ± 8.58	/
DP03	23.224.76*	/	/	73.54 ± 4.83*	/
DP04	6.152.29	/	/	63.73 ± 8.10*	/
DP05	39.727.78*	/	31.71 ± 9.00*	66.68 ± 5.90*	/
DP06	69.759.22*	61.21 ± 6.06*	63.53 ± 3.28*	86.42 ± 4.81*	62.07 ± 3.27*
UP07	5.461.09	/	/	24.86 ± 3.88	/
UP08	14.793.50*	/	/	26.85 ± 2.61	/
UP09	19.225.40*	/	/	36.92 ± 3.97*	/
UP10	4.982.21	/	/	31.59 ± 7.81*	/
UP11	16.254.67*	22.41 ± 1.72*	/	35.68 ± 3.82*	/
UP12	10.124.01	20.57 ± 4.30*	/	69.45 ± 5.97*	37.66 ± 5.24*
UP13	8.982.41	/	/	78.49 ± 3.39*	34.05 ± 6.12*
UP14	29.536.54*	27.56 ± 6.76*	12.88 ± 1.48*	57.28 ± 6.54*	/
UP15	18.963.89*	/	/	54.63 ± 4.19*	21.05 ± 5.28*

^1^Results represent the mean (± standard deviation SD). Each experiment was performed in triplicate. “/” represents the peptide completely degraded at the end. * Indicates a statistically significant difference when modified peptides were compared to Pep05 (*P* < 0.05 by ANOVA; *n* = 3).

### Cytotoxicity Against Human Erythrocytes and RAW 264.7 Cell

The cytotoxicity of all 16 peptides against human erythrocytes were determined in a hemolytic assay at peptide concentrations ranging between 32 and 128 μM. As shown in Table 9, peptides substituted with L- and D-amino acids displayed comparable hemolytic activities to Pep05, whereas those substituted with unnatural amino acids, with the exception of UP07, exhibited significantly increased hemolytic activities. These results suggest that peptides substituted with unnatural amino acids may be associated with an increased risk of hemolytic activity. Different observations were made with respect to the cytotoxicity of the peptides against the murine macrophage cell line RAW 264.7. Compared with Pep05, D-amino acid substituted peptides and UP09, UP11 and UP15 showed decreased cytotoxicity, while other unnatural amino acid substituted peptides showed similar cytotoxicity (Table 9).

**TABLE 9:** Cytotoxicity of Pep05 and derivatives against human erythrocytes and mouse macrophage RAW 264.7 cells.

Peptides	Hemolysis rate (%)^1^			IC_50_ value (μM) RAW 264.7
	32 μM	64 μM	128 μM	
Pep05	9.201.01	34.39 ± 1.49	49.18 ± 2.01	13.42 ± 2.85
LP01	7.660.46	29.58 ± 0.83	37.14 ± 1.29*	16.55 ± 3.79
LP02	9.150.37	24.18 ± 2.01	45.40 ± 2.40	14.43 ± 1.44
DP03	9.510.27	41.96 ± 4.14	51.55 ± 3.89	45.02 ± 5.96*
DP04	11.330.63	38.84 ± 1.61	58.26 ± 1.84	34.72 ± 3.69*
DP05	5.380.15	30.09 ± 1.56	45.07 ± 2.93	32.70 ± 3.44*
DP06	10.490.48	37.14 ± 2.73	59.99 ± 2.41	48.77 ± 6.97*
UP07	6.130.41	18.23 ± 2.76*	30.10 ± 2.61*	17.54 ± 2.99
UP08	16.231.45	52.49 ± 2.25*	86.84 ± 1.24*	14.56 ± 2.96
UP09	19.020.08*	55.99 ± 3.75*	95.99 ± 2.10*	26.21 ± 1.80*
UP10	22.521.07*	56.82 ± 2.62*	95.02 ± 1.84*	22.82 ± 4.65
UP11	70.732.66*	95.65 ± 2.31*	∼100.00*	28.34 ± 5.80*
UP12	23.491.15*	45.13 ± 1.95*	∼100.00*	21.24 ± 2.42
UP13	17.411.82*	41.03 ± 1.38	∼100.00*	21.74 ± 4.14
UP14	31.170.85*	66.51 ± 3.14*	74.68 ± 1.34*	13.97 ± 2.67
UP15	20.590.49*	54.16 ± 2.02*	97.86 ± 3.07*	28.69 ± 3.52*

^1^Results represent the mean (± standard deviation SD) of three independent experiments, each performed in triplicate. * Indicates a statistically significant difference when modified peptides were compared to Pep05 (*P* < 0.05 by ANOVA; *n* = 3).

### Evaluation of *in vivo* Toxicity

**FIGURE 6:**
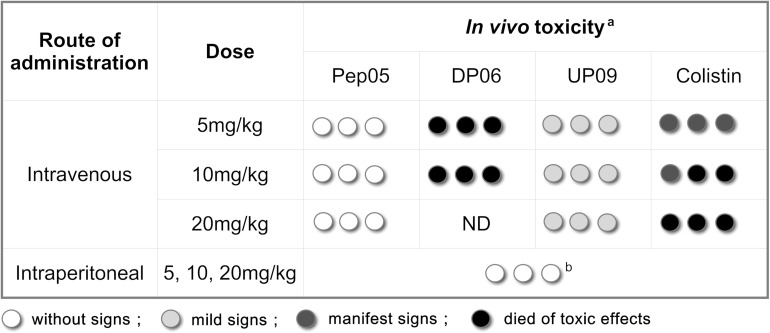
Acute *in vivo* toxicity of Pep05, DP06, UP09 or colistin at 5 mg/Kg, 10 mg/Kg and 20 mg/Kg. ^a^Three mice/group (each circle represents one mouse) were inoculated intravenously (i.v.) or intraperitoneally (i.p.) with Pep05, DP06, UP09, or colistin in a single dose and were monitored for 24 h. Different scales of gray indicate severity of signs as described in the legend. ^b^, No lethality and no signs of toxicity were observed for any of the tested peptides at doses of up to 20 mg/kg upon intraperitoneal injection.

*In vivo* toxicities of Pep05, DP06 and UP09 were evaluated based on a limited number of toxicity experiments. As summarized in Figure 6, a toxicity score was assigned as follows: no abnormalities = without signs; mild depression, poor motility, purple-black tail, and recovery in 1 h = mild signs; serious depression and poor motility even under stimulation = manifest signs; sudden death within 10 min = died of toxic effects. The mice intravenously injected with Pep05 displayed no obvious abnormalities at any of the tested doses. Mild depression and purple-black tails were observed for all of the tested intravenous doses of UP09, but recovered in 1 h. Strong toxicity was observed for intravenously administered DP06 at doses of 5 and 10 mg/kg, which induced sudden death within 1 min of injection (the dose of 20 mg/kg was not administered). Colistin also produced strong signs of toxicity, with two out of three mice dying immediately at 10 mg/kg and all three mice dying at 20 mg/kg. Upon intraperitoneal injection, no lethality and no signs of toxicity were observed for any of the tested peptides at doses of up to 20 mg/kg. All of the animals showed less than 5% weight loss at death or after 24 h. Hence, the replacement of L-Lys and L-Arg with the corresponding D-amino acids led to greater *in vivo* toxicity when the peptide was administered intravenously, despite the fact that this was not observed in the hemolytic activity and RAW 264.7 cytotoxicity assays. In contrast, UP09 displayed much lower *in vivo* toxicity than DP06 and colistin.

### Evaluation of *in vivo* Antibacterial Activities

**FIGURE 7:**
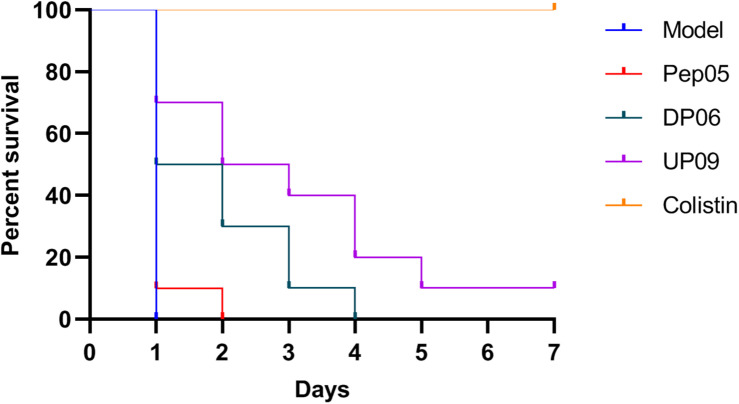
Kaplan–Meier survival curves for mice treated with Pep05, DP06, UP09, and colistin. Upon intraperitoneal administration of DP06, UP09, or colistin to mice infected with lethal doses of *P. aeruginosa*, the survival rates (*n* = 10) were 10%, 40%, and 100% after 3 d and 0%, 10%, and 100% after 7 days, respectively.

The MLD of *P. aeruginosa* ATCC 9027 was 5 × 10^6^ CFU/mouse. Upon intraperitoneal administration of DP06, UP09, or colistin to mice infected with lethal doses of *P. aeruginosa*, the survival rates (*n* = 10) were 10%, 40%, and 100% after 3 d and 0%, 10%, and 100% after 7 d, respectively (Figure 7). It should be noted that these experiments were conducted in a model of acute and rapidly progressing infection and that almost all of the animals in the untreated and Pep05 groups died within 24 h (survival rates of 0% and 10%, respectively). Thus, DP06 and UP09 still displayed improved potency, despite the relatively low absolute survival rates. It is interesting to note that UP09 exhibited better *in vivo* potency than DP06, despite the superior *in vitro* stability of the latter.

## Discussion

AMPs are considered promising anti-infective candidates for commercial and clinical applications owing to their high potency and selectivity, broad antimicrobial activity spectrum, low accumulation in tissues, and no propensity to trigger resistance (Costa et al., 2019). However, only a small fraction of the AMPs reported thus far have been approved for clinical use. Most of the approved AMPs are naturally derived from microorganisms and no linear AMPs composed entirely of natural L-amino acids have yet been approved for market. Most of the linear AMPs composed entirely of natural L-amino acids currently in phase II or III clinical trials are intended for external administration, such as omiganan for the treatment of rosacea and severe inflammatory acne vulgaris, LL37 for hard-to-heal venous leg ulcers, and pexiganan for diabetic foot ulcers (Costa et al., 2019). A key issue that must be addressed during the development of synthetic peptides as antimicrobial agents for systemic administration is identifying sequences that possess both resistance to proteolysis and selective toxicity (Oliva et al., 2018). In this regard, numerous strategies, such as peptide cyclization, side-chain modification, multimerization, drug delivery methods, and peptidomimetic approaches, have been found to display great potential for improving the *in vivo* application of synthetic AMPs (Ong et al., 2014). A useful approach for enhancing proteolytic stability is replacing the natural amino acids in AMPs with non-coded α-amino acid derivatives such as D- and unnatural amino acids (Kaminski and Feix, 2011; Di Grazia et al., 2015; Khara et al., 2016; Jia et al., 2017; Li W. et al., 2017; Qiu et al., 2017; Sun et al., 2017; Zaet et al., 2017; Oliva et al., 2018).

In this study, a series of peptide derivatives containing D- and unnatural amino acids were designed based on the previously reported cationic AMP Pep05. Dab and Dap are unnatural basic amino acids that occur in a variety of microbial peptide antibiotics, such as colistin, polymyxin B, and octapeptins (Velkov et al., 2010; Abou Fayad et al., 2018), among which colistin and polymyxin B are used as clinical anti-infective agents. Hor is another basic amino acid that possesses a very similar structure to L-Arg, with the only difference being an extra –CH_2_– group in the side chain. These unnatural amino acids can be used to replace the proteolytically sensitive L-Lys and L-Arg residues in AMPs while retaining the same net charge. Thi possesses a similar structure to phenylalanine. Aib is the simplest α,α-dialkyl amino acid and is a rare, non-proteinogenic amino acid that is only found in certain antibiotics of fungal origin with membrane permeation ability (Karle and Balaram, 1990; Yamaguchi et al., 2003; Zikou et al., 2007; Biondi et al., 2017). Although only a limited number of derivatives were examined in this study, various types, locations, and numbers of amino acid substitutions were investigated to achieve sequence diversity.

First, the physicochemical properties of the substituted AMPs, such as secondary structure and hydrophobicity, were investigated. Except for the series of D-amino acid derivatives, all of the peptides substituted with natural or unnatural L-amino acids maintained a helical structure. All amino acids except glycine exhibit chirality and helical antimicrobial peptides possess a topological structure, whereby peptides composed of L-amino acids always form right-handed helices, whereas those composed of D-amino acids always form left-handed helices. Substituting L-amino acids for D-amino acids therefore destroys the existing spiral topology (Garton et al., 2018). Our study also revealed that the helicity of the peptides decreased or even transformed into random coil in a hydrophobic environment (mimicked by 50% TFE) as the proportion of D-amino acids was increased, while the hydrophobicity decreased. As the physicochemical properties of L- and D-amino acids are very similar, the variation in hydrophobicity was presumably attributable to changes in the spatial structure.

To determine whether the amino acid substitutions definitively conferred increased resistance to proteases and to evaluate the proteolytic resistance of various amino acids, it is necessary to examine the degradation products and proteolytic degradation pathways in addition to measuring changes in the concentrations of the intact peptides. Therefore, in our study LC-MS was used to identify the degradation products of the modified AMPs in the presence of trypsin and plasma proteases. This is of great value for elucidating the degradation characteristics of AMPs and for introducing targeted modifications based on analysis of the protease sensitivities of various residues and positions.

Analysis of the degradation products revealed that both trypsin and plasma proteases tended to hydrolyze the peptides on the C-terminal sides of L-Lys and L-Arg, while the latter could also mediate hydrolysis on the C-terminal sides of hydrophobic residues such as L-Leu and L-Tyr. Trypsin is able to cleave after L-Lys and L-Arg residues at any position throughout a peptide chain, whereas plasma proteases predominantly hydrolyze peptides in the N-terminal region (the amidated C-terminal carboxyl groups of these peptides may have protected them from proteolytic degradation). Therefore, the peptides still retained partial activity after incubation with plasma as the degradation products were mainly large fragments that had lost only one or two residues at the N-terminus. The results clearly demonstrated that trypsin and the plasma proteases possessed much weaker hydrolytic activity toward D-Lys, D-Arg, L-Dab, L-Dap, and L-Hor than toward L-Lys and L-Arg. However, the presence of even one L-Lys or L-Arg residue in the peptide sequence led to instability because the protease could still mediate hydrolysis at this site (UP12 and UP15). Therefore, DP06, UP11, UP13, and UP14 displayed significantly increased resistance against trypsin and plasma proteases because all of the L-Lys and L-Arg residues in the original sequence had been replaced by their D-enantiomers or unnatural amino acids, among which DP06 exhibited the highest stability. Unfortunately, however, although the *in vitro* antibacterial activity and toxicity had not significantly changed compared with Pep05, DP06 displayed a complete loss of *in vivo* activity as well as severe toxicity. It remains unclear whether these effects are related to the change in secondary structure, i.e., the transformation to a random-coil structure in a hydrophobic environment, and further pharmacokinetic and toxicological studies are necessary to elucidate the underlying reason for these properties.

UP09 and UP10 were designed based on the replacement of the N-terminal L-Lys with Aib or Hor, respectively. Unexpectedly, in contrast to the other derivatives, UP09 displayed dramatically improved stability to plasma proteases despite the remaining L-Lys and L-Arg residues in the peptide sequence, whereas UP10 underwent rapid degradation in plasma. *In vivo* experiments also revealed that UP09 possessed improved biological activity compared with Pep05 and significantly reduced toxicity compared with DP06 and colistin. CD spectroscopy indicated that the secondary structure of UP09 was highly similar to that of Pep05 and, although the net positive charge was reduced, the other physicochemical properties did not change significantly, suggesting that the *in vivo* improvement of UP09 was not attributable to significant changes in the spatial structure or physicochemical properties. We speculate that the N-terminal Aib residue effectively protects this peptide from degradation by plasma proteases. As reported previously, Aib does not interact with the serine proteases elastase and thermolysin (Rival et al., 1999), and the introduction of Aib into a peptide backbone thus represents a useful strategy for enhancing its resistance toward proteases. Previous research has indicated that Aib tends to favor the formation of a helical conformation and this effect was reported to be more obvious when Aib is located in the inner region of the sequence (Conlon et al., 2007). Since the Aib replacement in UP09 occurred at the N terminus, it did not display significantly increased helicity through the CD analysis. Identification of the plasma degradation products demonstrated that the proteolytic degradation of the AMPs mainly occurred in the N-terminal region. Therefore, we speculate that the Aib residue protected UP09 from proteolysis mainly by inhibiting the binding of plasma proteases to the peptide. Further research to test this theory would be worthwhile.

## Conclusion

In conclusion, although the replacement of L-amino acids in biologically active peptides with D- or unnatural amino acids can effectively enhance proteolytic resistance, excessive modification or replacement is also associated with potential risks, such as cytotoxicity, immunogenicity, and other toxic effects. For example, in our study Pep05 displayed no obvious toxicity, whereas DP06, with all of the original L-Lys and L-Arg residues replaced with their D-amino acids exhibited very severe *in vivo* toxicity. In addition, the cost of synthesizing peptides containing D- and unnatural amino acids is higher than that for peptides composed solely of L-amino acids. Therefore, the probability of natural peptides being developed into drugs will be increased if fewer modifications are used to improve the *in vivo* stability, maintain the biological activity, and not significantly increase the toxicity, as in the case of UP09. Although UP09 displayed improved antibacterial activity compared with Pep05 and DP06 in the *in vivo* antimicrobial activity tests, this activity was still much weaker than that of colistin, which possesses very strong anti-gram-negative bacteria activity in clinical use. However, the clinical applications of colistin are limited by its pronounced nephrotoxicity and neurotoxicity (Falagas and Kasiakou, 2006), and colistin-resistant strains have frequently been isolated in the clinic (Srinivas and Rivard, 2017). In contrast, UP09 exhibits broad-spectrum antimicrobial activity in addition to much lower *in vivo* toxicity than colistin on the basis of the preliminary toxicity evaluation performed in this study, and is less prone to trigger resistance by targeting the microbial membrane. Therefore, UP09 may be a potential lead compound for further optimization to obtain antimicrobial agents with superior *in vivo* activity and safety. The discovery of UP09 also provides a new strategy for the design of novel AMPs to overcome low bioavailability.

## Data Availability Statement

The raw data supporting the conclusions of this article will be made available by the authors, without undue reservation.

## Ethics Statement

The animal study was reviewed and approved by Ethical Committee of Experimental Animals of the Chia Tai Tianqing Pharmaceutical Group.

## Author Contributions

JL and JXi carried out design, chemical peptide synthesis and molecular weight confirmation of all AMPs. JL and JXu contributed to the hydrophobicity and protease stability assessments (HPLC and LC-MS assays). HX designed and carried out the *in vivo* antimicrobial activity and toxicity assays. JM, JL, and YL carried out the *in vitro* antimicrobial activity, hemolysis and cytotoxicity assays. JF supervised all work. All authors contributed and approved the final revision of the manuscript.

## Conflict of Interest

JL, JM, JXu, and JF were employed by the company Shanghai Duomirui Biotechnology Co., Ltd. HX was employed by the company Chia Tai Tianqing Pharmaceutical Group Co., Ltd. The remaining authors declare that the research was conducted in the absence of any commercial or financial relationships that could be construed as a potential conflict of interest.
