# Supplementary material for: D- and Unnatural Amino Acid Substituted Antimicrobial Peptides With Improved Proteolytic Resistance and Their Proteolytic Degradation Characteristics
Source: Front Microbiol. 2020 Nov 12;11:563030. doi: 10.3389/fmicb.2020.563030 (PMC7688903; doi:10.3389/fmicb.2020.563030)
Supplement: Supplementary file 1 [file Data_Sheet_1.docx]

Supplementary Material

# Supplementary Figures and Tables

## Supplementary Figures

|   Pep05 |   LP01 |   LP02 |
| --- | --- | --- |
|   DP03 |   DP04 |   DP05 |
|   DP06 |   UP07 |   UP08 |
|   UP09 |   UP10 |   UP11 |
|   UP12 |   UP13 |   UP14 |
|   UP15 |  |  |

**Supplementary Figure 1.** ESI(+)-MS spectra of prepared Pep05 and derivatives.


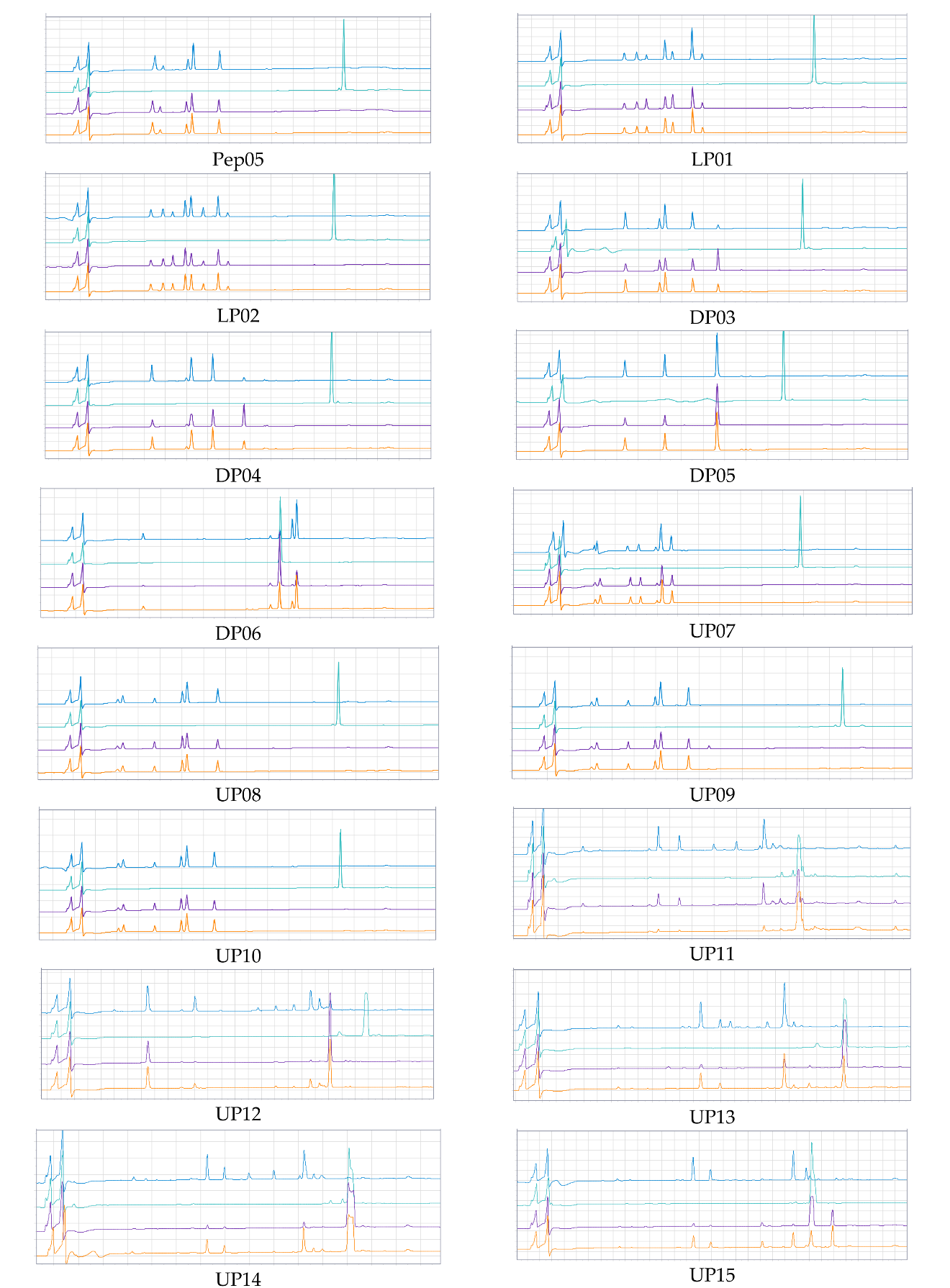


**Supplementary Figure 2** Stabilities of Pep05 and derivatives towards trypsin monitored by HPLC. Chromatograms of each reaction at different time points are overlapped for comparison. The turquoise (”**──**”) chromatogram represents the negative control without trypsin and the main peak represents the peptide at 0h. The purple (”**──**”) chromatogram represents 1h, the orange (”**──**”) represents 5h and the blue (”**──**”) represents 18h.


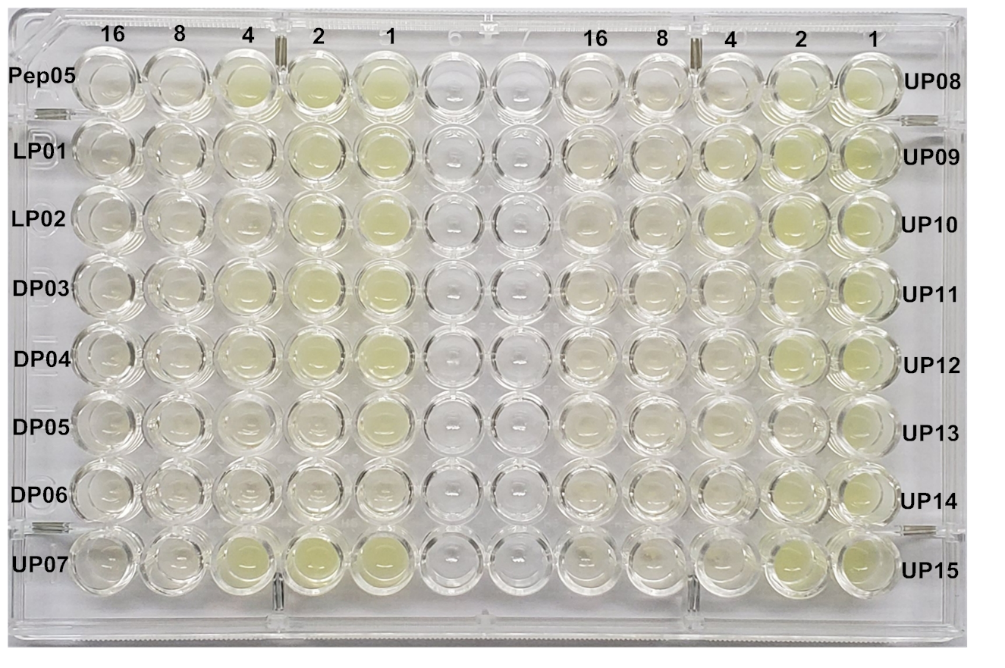


**Supplementary Figure 3** Antimicrobial activities of Pep05 and its derivatives after incubation with human plasma. Each peptide was tested at five concentrations ranging from 1- to 16-fold MIC. The values at the top of the 96-well plate represent multiples of the concentration relative to the respective MIC. A change in color from colorless to green indicated microorganism growth.


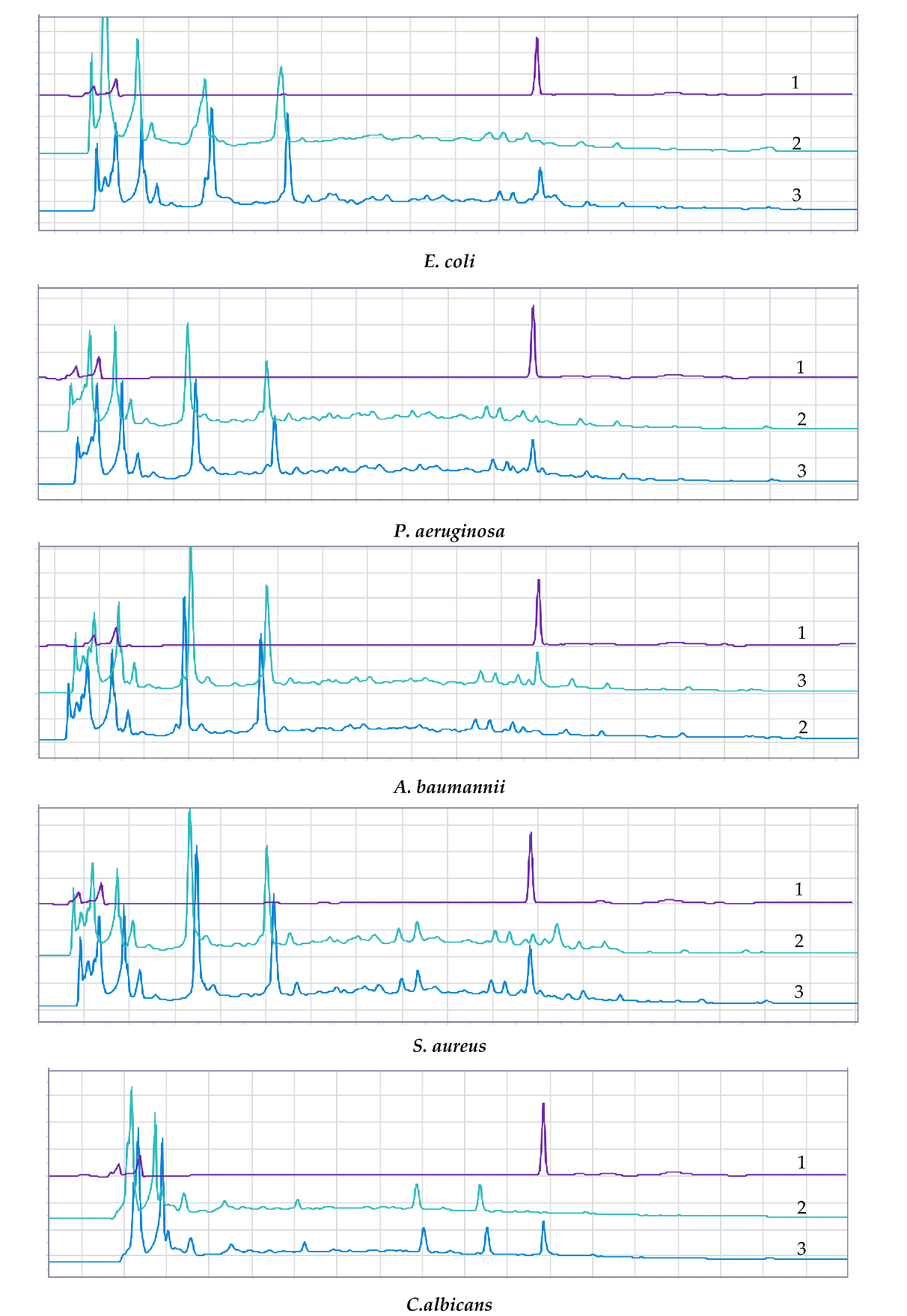


**Supplementary Figure 4** Stability of DP06 toward proteases secreted by various bacterial strains. DP06 was incubated with supernatants from cultures of (A) *E. coli*, (B) *P. aeruginosa*, (C) *A. baumannii*, (D) *S. aureus*, and (E) *C. albicans* for 24 h. The peptide stability was then determined by HPLC. Chromatograms 1 correspond to the peptide only, chromatograms 2 correspond to the supernatant only, and chromatograms 3 correspond to the mixed supernatant and peptide.
